# Associations of preschool reactive bed-sharing with sociodemographic factors, sleep disturbance, and psychopathology

**DOI:** 10.1186/s13034-023-00607-w

**Published:** 2023-05-17

**Authors:** Susan E. Marakovitz, R. Christopher Sheldrick, William E. Copeland, Bibiana Restrepo, Ingrid Hastedt, Kimberly L.H. Carpenter, Ellen W. McGinnis, Helen L. Egger

**Affiliations:** 1 Brigham and Women’s Hospital, Department of Pediatric Newborn Medicine, Boston, MA USA; 2 Department of Health Law, Policy & Management, School of Public Health, Boston University, Boston, MA USA; 3 Vermont Center for Children, Youth and Families, University of Vermont, 1 S. Prospect Street, Burlington, VT 05401 USA; 4 UC Davis Medical Center, Sacramento, CA USA; 5 Department of Psychology, University of Massachusetts Boston, Boston, MA USA; 6 Department of Psychiatry and Behavioral Sciences, Duke University School of Medicine, Durham, NC USA; 7 Department of Child and Adolescent Psychiatry, Hassenfeld Children’s Hopsital at NYU Langone, New York, NY USA

**Keywords:** Bed-sharing, Prevalence, Preschool, Sleep problems, Anxiety, Disruptive behavior, Psychopathology

## Abstract

**Objective:**

To advance understanding of early childhood bed-sharing and its clinical significance, we examined reactive bed-sharing rates, sociodemographic correlates, persistence, and concurrent and longitudinal associations with sleep disturbances and psychopathology.

**Methods:**

Data from a representative cohort of 917 children (mean age 3.8 years) recruited from primary pediatric clinics in a Southeastern city for a preschool anxiety study were used. Sociodemographics and diagnostic classifications for sleep disturbances and psychopathology were obtained using the Preschool Age Psychiatric Assessment (PAPA), a structured diagnostic interview administered to caregivers. A subsample of 187 children was re-assessed approximately 24.7 months after the initial PAPA interview.

**Results:**

Reactive bed-sharing was reported by 38.4% of parents, 22.9% nightly and 15.5% weekly, and declined with age. At follow-up, 48.9% of nightly bed-sharers and 88.7% of weekly bed-sharers were no longer bed-sharing. Sociodemographics associated with nightly bed-sharing were Black and (combined) American Indian, Alaska Native and Asian race and ethnicity, low income and parent education less than high school. Concurrently, bed-sharing nightly was associated with separation anxiety and sleep terrors; bed-sharing weekly was associated with sleep terrors and difficulty staying asleep. No longitudinal associations were found between reactive bed-sharing and sleep disturbances or psychopathology after controlling for sociodemographics, baseline status of the outcome and time between interviews.

**Conclusions:**

Reactive bed-sharing is relatively common among preschoolers, varies significantly by sociodemographic factors, declines during the preschool years and is more persistent among nightly than weekly bed-sharers. Reactive bed-sharing may be an indicator of sleep disturbances and/or anxiety but there is no evidence that bed-sharing is an antecedent or consequence of sleep disturbances or psychopathology.

**Supplementary Information:**

The online version contains supplementary material available at 10.1186/s13034-023-00607-w.

Bed-sharing, defined as a child and parent sleeping in the same bed for part or all of some or all nights, is a practice that has varied widely around the world and throughout human history [1]. In the United States (US), bed-sharing has been discouraged due to evidence linking this sleep practice with Sudden Infant Death Syndrome. Additionally, some pediatric professionals have argued that it negatively impacts the developing child by interfering with self-regulation and autonomy, and possibly contributes to sleep and mental health problems [2]. The American Academy of Pediatrics (AAP) recommends against bed-sharing for at least six months and ideally through the first year of life [3], but offers no recommendations for older children. Despite the AAP recommendations, bed-sharing during infancy has been on the rise in the US [4]. Beyond infancy, less is known about bed-sharing prevalence, developmental trends and whether this sleep practice is associated with clinically significant problems. This information gap makes it difficult for pediatric professionals to address parental concerns or provide evidence-based guidance after infancy.

Although prevalence and developmental trends in US bed-sharing during early childhood are not well-characterized, several themes emerge across available studies. First, bed-sharing in early childhood is relatively common. In the 1980 and 1990 s, several studies found 41–55% of children between the ages of 6 and 48 months shared a bed with a parent at least occasionally [5–7]. Second, consistent bed-sharing, defined as several times a week or more, occurs in roughly 25% of families [6, 8, 9]. Third, bed-sharing appears to be influenced by sociodemographic factors. In early childhood, it has been reported that US bed-sharing rates are higher in Black, Hispanic, and mixed race families than White families [5–8, 10]. Beyond infancy, very little is known about US bed-sharing rates in families across different n racial and ethnic groups, particularly among those of Asian, American Indian or mixed race backgrounds. Additionally, there are reports that bed-sharing is more commonly practiced among families with lower socioeconomic status and parental education, and in families with a single mother as head of household [4–6, 10, 11]. These results were found more consistently in large, representative US samples [4, 10] than in smaller, predominantly low income [8], urban [5] and Hispanic [7] samples. Finally, bed-sharing varies significantly with child age. Cross-sectional and longitudinal studies from the US and Europe indicate that bed-sharing increases after the first year of life [7, 12], remains relatively stable from 1 to 3 years of age [6, 9, 12] and declines after 4 or 5 years of age [11, 12].

Parents and pediatric healthcare professionals may question whether bed-sharing is associated with developmental risk or harm after infancy. Multiple studies have documented concurrent associations between bed-sharing and sleep problems, particularly night waking and bedtime resistance [5, 6, 13–15]. There is some - albeit weak - evidence of a concurrent link between bed-sharing and behavior problems in a mixed-age clinical sample (2–13 years) [16] and generalized anxiety disorder in a Turkish adolescent sample using retrospective reporting [17]. In prospective studies with non-clinical, preschool samples, results are mixed. Several studies have documented longitudinal associations between bed-sharing and sleep problems [6, 12, 18] whereas only one study [19] out of five [6, 8, 11, 19, 20] demonstrated associations with psychopathology, specifically overall psychopathology, anxiety and depression.

Combining key bed-sharing subtypes may contribute to these mixed findings. Conceptual models of bedsharing [21–23] distinguish between *intentional* bed-sharing that is chosen proactively by parents due to parenting preferences and/or cultural norms and *reactive* bed-sharing that is a response to factors such as child behavior or limited resources [6, 24]. In comparison to intentional bed-sharing, reactive bed-sharing has been associated with a higher rate of sleep problems such as night wakings [25] and may have stronger associations with child behaviors such as refusal to sleep alone [6], night-time fears, nightmares, bedtime resistance/tantrums or clinical problems such as separation anxiety, hyperactivity or oppositionality.

Methodological limitations also contribute to the mixed findings. First, most studies have assessed bed-sharing using a single questionnaire or interview item [7, 8, 26], or multiple items without measureing reliability [6, 11]. Direct observations of bed-sharing are small and rare and often recruit based on bed-sharing status, thus concurrent validity with other assessment are not available [13, 27]. Additionally, most studies have used parent-report questionnaires to assess sleep and mental health problems rather than structured parent interviews that generate diagnostic classifications. Reliance on questionnaires may have obscured meaningful associations because children with elevated problems and those with clinically significant disorders were combined. Furthermore, when longitudinal associations have been identified, investigators have not controlled for baseline symptomatology [19] or assessed bidirectionality consistently. Thus, it is unknown whether bed-sharing was a result of child anxiety and depression or associated with their emergence. Additionally, These methodological gaps limit guidance that can be provided to parents and clinicians working with families.

To advance understanding of early childhood bed-sharing (after infancy) and whether it is a cause for concern in the US, we focused on reactive bed-sharing and examined (1) prevalence and associated sociodemographic factors, (2) developmental trends, and (3) concurrent and longitudinal associations between bed-sharing and sleep disturbances and mental health diagnoses. We explored sociodemographics correlates (race, ethnicity, socioeconomic factors) of reactive bed-sharing to provide preliminary information about potential cultural variation in bed-sharing and to inform directions for future research on culturally-sensitive family sleep guidance. In our longitudinal analyses, we addressed prior methodological weaknesses by controlling for baseline symptomatology and exploring directionality.

## Method

### Study design

The Duke Preschool Anxiety Study is a screen-stratified study of 917 preschool children recruited from Central North Carolina primary care pediatric clinics. The original aims of the study were to characterize the prevalence, comorbidities and risk factors of preschool anxiety disorders. Therefore, children who screened high for anxiety were over-sampled, a procedure used to ensure that an adequately representative sample of children with anxiety disorders was obtained to address the primary goals of the project. To establish prevalence rates from a multistage sampling design, subjects were assigned a weight inversely proportional to their probability of selection. The original study was approved by Duke University School of Medicine Institutional Review Board (IRB).

### Procedures

Children ages 2 through 5 years attending the Duke Children’s Pediatric Primary Care Clinics were screened for study eligibility from January of 2007 to October of 2010. Of the 3433 children screened, 943 (27.5%) screened high and 2490 (72.5%) did not screen high for anxiety using the Child Behavior Checklist (CBCL) for Ages 1½–5 anxious/depressed scale. A cutpoint based on data from an earlier study [28] was used as a benchmark to identify a group consisting of approximately 25% of the primary care clinic sample who were at relatively high risk of having an anxiety disorder. The cutpoint was adjusted during the study to ensure that the correct proportion of participants (25%) were being identified (cutpoint of 4 was used for 0.3% of the cohort, 5 for 62.0%, and 7 for 37.7%). All of the children who screened high and 189 (7.5%) who did not screen high were selected to participate.

Of the 1132 children selected to participate, 1113 were eligible. Inclusion criteria for screening were (1) the child was 24–71-months-old, (2) attended the clinic during the screening period, and (3) a parent/legal guardian was present and consented to screening. Exclusion criteria were (1) lack of a parent/legal guardian with adequate fluency in English, (2) the child was known to have intellectual disability (IQ < 70), autism, or other pervasive developmental disorders, (3) the child’s sibling was already participating, (4) the child was not accompanied by a legal guardian who could provide consent, or (5) the child was considered by the provider to be too medically ill on the day of screening to participate. Nineteen (1.7%) met exclusion criteria and 196 (17.6%) parents/legal guardians chose not to participate. Informed consent forms were signed by a parent or legal guardian by 917 (82.4%) of eligible parents prior to each phase of the study including screening, and interviews. PAPA assessments took place over 47 months (January 2007–December 2010).

A follow-up assessment was completed with 187 of the original participants as part of a neuroimaging study. Children with anxiety disorders were oversampled (representing two-thirds of the cohort), and the remainder of the sample were healthy controls from the original cohort. Informed consents were signed and PAPA interviews were completed again approximately 24.7 months after initial assessment. More detail on study design is available in previous publications [29–31].

### Measures

Preschool Age Psychiatric Assessment (PAPA)^[[32]]^. The PAPA, a parent-report interview that assesses psychopathology in 2- to 5-year-olds, is based on the parent version of the Child and Adolescent Psychiatric Assessment [33]. The PAPA uses a highly structured protocol, with required questions and probes. However, the interviewer plays a key role by ensuring that interviewees understand the question being asked, provide clear information on behaviors or feelings relevant to the symptom, and report the symptom at a prespecified level of severity as defined in an extensive glossary. Interviewers were certified by a qualified PAPA trainer after 2 weeks of classroom didactics, 4 on-study interviews with live trainer observation, 10 on-study interviews with trainer video review and feedback. All study interviews were checked for fidelity, and weekly random selected interviewers were reviewed by the entire study team to insure against interviewer drift and fidelity of coding. Test-retest reliability of the PAPA is on a par with those achieved by older child, adolescent, and adult psychiatric interviews [28].

The PAPA evaluates diagnoses drawn primarily from the Diagnostic and Statistical Manual of Mental Disorders, Fourth Edition but also other diagnostic classification systems applicable to young children such as the Research Diagnostic Criteria: Infancy and Preschool [34]. Once the interviewer determines that a symptom is present based on a pre-established definition, frequency, duration, and dates of onset are collected. A 3-month “primary period” is used because shorter recall periods are associated with more accurate recall [33]. Diagnoses and symptom counts for the following disorders and difficulties were the focus in this study: (1) depressive disorders including major depression, dysthymia and depressive disorder, not otherwise specified, (2) anxiety disorders including separation anxiety, general anxiety, social phobia and specific phobia, (3) disruptive behavior disorders including attention-deficit/hyperactivity disorder, oppositional defiant disorder and conduct disorder, and (4) sleep disturbances including difficulty initiating sleep, difficulty maintaining sleep, sleep terrors, nightmares, and sleep walking. Impairment was based on the World Health Organization’s International Classification of Functioning, Disability and Health [35].

A bed-sharing variable was created using a series of questions in the sleep section of the PAPA. Parents were first asked whether their child slept with a family member for part of the night or the whole night due to reluctance to sleep alone. If this behavior was present, frequency, duration and onset were obtained. Frequency of bed-sharing was used to create three categories: (1) None/Rare: no bed-sharing or rare bed-sharing, 2) Weekly: bed-sharing at least once per week but less than every night, and 3) Nightly: bed-sharing every night. Parental reported, interview based assessments of bed-sharing and classification by bed-sharing frequency are consistent with prior literature [6, 7, 11]. All participants provided a coded response to the question about bed-sharing. Test-retest reliability for bedsharing frequency and subsequent classifications were good (r(300) = 0.81 and 0.79) for the 300 families administered the PAPA again about 11 days following the first interview [28].

Sociodemographic information, obtained from the background section of the PAPA,was also examined including child age, sex and race and ethnicity, parental education level, and poverty. Race and ethnicity was categorized as follows: Black/African American, Hispanic, White, and (combined) Asian, American Indian, Alaska native, Native Hawaiian, Other Pacific Islander, and 2 or more races. Caregivers could mark off all categories applicable to the child. The assessment of poverty was determined by referencing the family’s reported income level against the annual US federal guidelines for poverty thresholds given the number of individuals in the family [36].

### Statistical analyses

All analyses were conducted using weighted logistic and poisson regression in the SAS Version 9 Software procedure GENMOD. Odds ratios are provided for dichotomous outcome variables and means ratios for Poisson distributed scales. To account for sampling procedures, participants from all samples were assigned a weight inversely proportional to their probability of selection to account for screen-stratification. Thus, results from all analyses reported here are intended to represent unbiased estimates for the original primary care population from which the sample was drawn. Sandwich type variance corrections were applied to adjust for the parameter and variance effects induced by the sampling stratification.

Bivariate analyses involved prediction of sociodemographic and psychopathology variables by dummy-coded independent variables representing each frequency of bed-sharing (i.e., None/Rare, Weekly, or Nightly). For analyses predicting psychopathology status, models were adjusted for significant covariates including age, parental education, race and ethnicity, family poverty status and other diagnoses. In parallel analyses, we substituted dummy variables for psychopathology with counts of symptoms in each category. Longitudinal analyses were adjusted for a similar set of covariates as well as time since initial interview and status of outcome variable at baseline.

## Results

Bed-sharing rates and associations with sociodemographic factors are presented in Table 1. Values in Table 1 are adjusted for sampling and describe differences across three levels of bed-sharing frequency (None/Rarely, Weekly or More, Nightly). Our sample was diverse, with weighted percentages indicating that 53.1% reported minority race and ethnicity and 11.9% reported family income below the federal poverty level. The average age of participants at initial assessment was 3.8 years and there was no difference by sex. Bed-sharing was relatively common in our sample. Parents reported that 38.4% of children were bed-sharing, and 22.9% occurred nightly. Nightly bed-sharing was more common in families reporting child race and ethnicity as Black and combined American Indian, Alaska Native, or Asian compared to White (Hispanic and Non-Hispanic), with income below compared to above the federal poverty threshold, and in families with parents who obtained less than a high school education compared to those with education beyond high school. Weekly bed-sharing was more common in families reporting child race and ethnicity as Black compared to White.

**Table 1:** Associations between bed-sharing status and sociodemographic factors

	None/Rarely	Weekly	Nightly	Nightly vs. none	Weekly vs. none
	%(n)	%(n)	%(n)	OR (95%CI), p	OR (95%CI), p
Total	61.5 (453)	15.5 (169)	22.9 (295)		
Age	4.0 (1.3)	3.8 (1.3)	3.6 (1.2)	**0.6 (0.4–0.8), 0.003**	**0.6 (0.4–0.9), 0.01**
% Male	52.9 (229)	48.9 (84)	50.7 (153)	1.1 (0.6–1.9)	1.2 (0.6–2.3)
% Hispanic	14.7 (49)	7.9 (19)	11.1 (36)	1.3 (0.5–3.4), 0.55	0.7 (0.2–2.4), 0.60
% Black	25.8 (168)	42.8 (74)	42.6 (142)	**2.9 (1.5–5.5), 0.001**	**2.3 (1.1–4.7), 0.03**
% White	54.4 (204)	40.2 (62)	31.1 (81)	--	--
% American Indian, Alaska Native or Asian	5.1 (32)	9.1 (14)	15.1 (36)	**5.1 (1.9–14.1), 0.002**	2.4 (0.7–8.7), 0.18
Family Income					
Below Poverty	10.6 (76)	7.7 (22)	18.5 (70)	**1.9 (1.0-3.9), 0.05**	0.7 (0.3–1.8), 0.48
Highest Education					
Less than HS	4.4 (36)	2.3 (10)	12.0 (47)	**3.4 (1.3–8.8), 0.01**	0.5 (0.2–1.3), 0.12
HS grad	12.3 (70)	11.9 (21)	13.5 (46)	1.4 (0.6-3.0), 0.45	0.9 (0.3–2.3), 0.74
Some college	23.2 (116)	17.3 (55)	26.2 (91)	1.4 (0.7–2.7), 0.31	0.7 (0.3–1.4), 0.25
College grad.	60.1 (230)	68.5 (82)	48.3 (110)	--	--

### Concurrent associations with sleep disturbances and psychopathology

After controlling for sociodemographics and other forms of psychopathology, multivariate ordinal logistic regression analyses indicated that only anxiety disorder status was associated with nightly bed-sharing and only sleep disturbance status was associated with weekly bed-sharing. Parallel analyses with continuous symptom variables revealed the same pattern. These results are depicted in Table 2.

**Table 2:** Associations of bed-sharing status with diagnostic categories adjusted for age, sex, parental education, family poverty status, and other diagnoses

	Nightly vs. none	Weekly vs. none
	OR (95%CI), p	OR (95%CI), p
**Diagnostic Categories**		
Depression	1.0 (0.4–2.4), 0.97	0.7 (0.3–2.1), 0.56
Any Anxiety	**3.4 (1.7–6.8), < 0.001**	0.6 (0.3–1.2), 0.12
Any Disruptive Behavior	0.5 (0.2-1.0), 0.06	1.6 (0.6–4.3), 0.37
Any Sleep Disturbance	2.0 (0.9–4.8), 0.12	**2.5 (1.1–5.7), 0.03**
**Symptoms**		
Depression	1.2 (0.9–1.5), 0.26	1.1 (0.9–1.5), 0.38
Anxiety	**1.5 (1.3–1.7), < 0.01**	1.0 (0.9–1.3), 0.72
Disruptive Behavior	0.9 (0.8–1.2), 0.54	0.9 (0.7–1.1), 0.36
Sleep Disturbance	1.4 (0.9–2.5), 0.22	**2.2 (1.1–4.5), 0.03**

Table 2 Note: All analyses herein were adjusted for sociodemographic variables from Table 1. To further explore these results, we analyzed associations between bed-sharing and specific types of anxiety disorders and sleep disturbances. Among anxiety disorders, separation anxiety disorder displayed the strongest association with nightly bed-sharing. Among sleep disturbances, sleep terrors were associated with weekly and nightly bed-sharing whereas difficulty staying asleep was associated with weekly bed-sharing. These results are presented in Table 3.

**Table 3:** Associations of bed-sharing status with diagnostic categories adjusted for age, sex parental education and family poverty status

	Nightly vs. none	Weekly vs. none
	OR (95%CI), p	OR (95%CI), p
**Anxiety Diagnosis**	**3.2 (1.6–6.1), < 0.001**	0.7 (0.3–1.3), 0.22
Separation Anxiety Disorder	**7.8 (3.3–18.4), < 0.001**	1.4 (0.6–3.3), 0.45
Specific Phobia	1.1 (0.5–2.5), 0.73	0.6 (0.2–1.4), 0.22
Social Phobia	1.3 (0.7–2.5), 0.44	0.9 (0.4–1.9), 0.71
Generalized Anxiety Disorder	1.7 (0.6–5.1), 0.34	1.6 (0.5–5.5), 0.45
**Sleep Disturbance**	**2.3 (1.0-5.1), 0.04**	**2.3 (1.0-5.3), 0.04**
Initiating sleep	1.8 (0.6–5.5), 0.32	2.5 (0.9–7.1), 0.10
Staying asleep	4.1 (0.8–20.2), 0.08	**4.4 (1.2–15.9), 0.03**
Sleep terrors	**4.1 (1.2–13.8), 0.02**	**3.5 (1.0-11.8), 0.04**
Nightmares	0.7 (0.3–2.1), 0.57	2.3 (0.5–11.1), 0.32

Note: Sleep walking was included in the sleep disorder group but too few cases were available to test individual associations. All analyses herein were adjusted for sociodemographic variables from Table 1

### Bed-sharing persistence

Among those who reported nightly bed-sharing at baseline, only 41.3% continued to report nightly bed-sharing at follow-up, while 9.7% reported less frequent bed-sharing (at least weekly) and 48.9% reported no bed-sharing. Thus, nightly bed-sharing declined but did not disappear. In contrast, weekly bed-sharing at baseline declined markedly to the point that 88.7% reported no bed-sharing at all at follow-up. These results are very similar to rates with no bed-sharing at baseline, as 89.9% of these parents continued to report no bed-sharing at follow-up. Overall, the odds of nightly bed-sharing at follow-up were 32 times higher (95% CI: 6.5–158,3, p < 0.001) if nightly bed-sharing was reported at baseline compared to no bed-sharing. Bed-sharing was not associated with presence of follow-up visit (*p* = 0.26).

### Longitudinal associations with sleep disturbances and psychopathology symptoms

We also examined bidirectional longitudinal associations between nightly bed-sharing and sleep disturbances as well as psychopathology symptoms (see Table 4). Baseline nightly bed-sharing was associated with total symptom count, depression symptoms and anxiety symptoms but not disruptive behavior or sleep disturbance symptoms at follow-up. None of these associations were significant, however, after adjusting for baseline symptom status, time since the initial interview and sociodemographics. A similar set of models tested whether early symptoms were associated with bed-sharing at follow-up. Here again, despite evidence of longitutindal associations from baseline total and anxiety symptoms to later bed-sharing, all associations attenuated when models were adjusted for baseline bed-sharing and other covariates.

**Table 4:** Bidirectional longitudinal associations between nightly bed-sharing and symptom count

		Unadjusted		Adjusted	
Predictor (wave 1)	Outcome	OR (95% CI)	*p*	OR (95% CI)	*p*
Bed-sharing	Total symptom	**1.5 (1.0-2.2)**	**0.05**	1.1 (0.9–1.5)	0.38
Bed-sharing	Depression sx.	**2.0 (1.1–3.4)**	**0.02**	1.4 (0.9–2.2)	0.11
Bed-sharing	Anxiety sx.	**1.6 (1.1–2.3)**	**0.02**	1.1 (0.8–1.6)	0.50
Bed-sharing	DBD sx.	1.5 (0.9–2.5)	0.17	1.2 (0.8–1.8)	0.40
Bed-sharing	Sleep disturbance	2.5 (0.5–13.0)	0.29	4.5 (0.9–22.3)	0.06
Any symptom	Bed-sharing	**1.1 (1.0-1.1)**	**0.01**	1.1 (1.0-1.1)	0.14
Depression sx.	Bed-sharing	1.3 (0.9–1.7)	0.15	1.1 (0.8–1.7)	0.50
Anxiety sx.	Bed-sharing	**1.3 (1.1–1.4)**	**< 0.001**	1.1 (0.9–1.4)	0.21
DBD sx.	Bed-sharing	1.1 (1.0-1.2)	0.07	1.1 (1.0-1.2)	0.16
Sleep problems	Bed-sharing	1.9 (0.8–4.5)	0.14	1.6 (0.8–3.1)	0.20

Note: DBD = Disruptive Behavior Disorder, sx.= symptoms. All analyses herein were adjusted for sociodemographic variables from Table 1, and time since the previous assessment

## Discussion

Bed-sharing due to child reluctance to sleep independently (i.e. reactive bed-sharing) was a common practice in early childhood with over 1 in 3 parents reporting some bed-sharing and over 1 in 5 reporting nightly bed-sharing. However, prevalence varied significantly by sociodemographic factors, with higher rates in families characterized with lower income and educational attainment, and in families reporting child race and ethnicity of Black and (combined) American Indian, Alaska Native and Asian. Bed-sharing prevalence also decreased over development with lower rates among older compared to younger preschoolers. Cross-sectional associations suggested higher levels of separation anxiety and sleep terrors in children who bed-share nightly, and difficulty maintaining sleep and sleep terrors in children who bed-share weekly. Similar associations were observed in longitudinal models. However, associations attenuated after controlling for sociodemographics, time between interviews, and baseline status of the outcome. Our findings suggest reactive bed-sharing is a common practice in families with young children that varies by sociodemographic factors, declines over time, and is a more meaningful indicator of current problems than a risk factor for later struggles.

Our overall reactive bed-sharing prevalence was slightly lower than the 41–55% reported in some previous studies [5–7], but quite similar to the 38% bed-sharing prevelance reported for 4-year-old Swiss children in a 10-year longitudinal study of bed-sharing [12], and the 22% nightly bed-sharing prevalence reported for 3–5 year olds in the United States [37]. We also found a higher percentage of families were bed-sharing nightly than weekly, a result which runs counter to prior reports that intermittent bed-sharing is more common than frequent bed-sharing [6, 8, 9]. The higher rates of nightly than weekly bed-sharing might be due to the diverse nature of our sample and/or emphasis on reactive bed-sharing.

Sociodemographic factors were associated with bed-sharing. Consistent with previous research [4, 6, 10, 11], bed-sharing was more common among families reporting low socioeconomic status such as income below the poverty threshold and educational attainment less than high school. Bed-sharing was also more common in families of Black and (combined) American Indian, Alaska Native and Asian, but not Hispanic, race and ethnicity. Due to our small sample sizes of American Indian, Alaska Native and Asian families, we cannot make any conclusions about these groups individually. Altogether the differences suggest a need to better understand whether potential variation in family cultural beliefs, values, and norms about child sleep reluctance influence bed-sharing prevalence. These findings are consistent with prior work documenting higher bed-sharing rates for any reason for those reporting Black race and ethnicity [5, 6, 28] and add to the mixed results for Hispanic race and ethnicity [7, 8, 10]. These sociodemographic correlates of bed-sharing suggest, as argued by other investigators [12, 38], that this sleep practice is influenced by an interplay of child reluctance, family/parenting and cultural/subcultural factors. Although our assessment explicitly inquired about reactive bed-sharing due to child reluctance to sleep alone, some families in our sample may have been bed-sharing for additional reasons including bed/crib availability, warmth, and protection against neighborhood violence [39, 40] and as a way to preserve a sense of culture and/or as a family tradition which was practiced by parents and grandparents [40, 41]. These reasons for bed-sharing were not assessed in the current study, and further research is needed to better understand additional reasons for bed-sharing. However, taken together, there is likely not a one-size-fits-all recommendation about normative or problematic bed-sharing when working with families from diverse backgrounds.

We were also interested in the clinical significance of preschool-age bed-sharing. At baseline adjusting for co-morbidities, weekly bed-sharing was associated with sleep terrors and difficulty staying asleep, and nightly bed-sharing was associated with separation anxiety and sleep terrors. Our findings are consistent with prior work indicating a fairly robust concurrent link between bed-sharing and sleep problems [5–7, 13, 14] and negligible support for associations with internalizing and externalizing disorders [6, 16, 17]. To our knowledge, no prior investigations have linked bed-sharing to separation anxiety or sleep terrors despite the high prevalence of these disorders during early childhood. Our ability to detect these disorder-specific associations may be due to our assessment method (e.g., structured diagnostic interview versus parent-report questionnaires) and multiple bed-sharing definitions (e.g., weekly and nightly).

Cross-sectional examination of bed-sharing does not address developmental trends. In our sample, bed-sharing decreased but did not disappear from the initial assessment when children were on average 3.8 years old to the follow-up assessment approximately 2 years later. At follow-up, only 41.3% of parents who initially reported nightly bed-sharing and only 11.3% of parents who initially reported weekly bed-sharing continued to do so. These findings are commensurate with previous research indicating that bed-sharing increases after infancy [7], is modestly stable from one to three years of age [6, 9, 28] and, then, starts to decline around 4 ^12^ or 5 ^11^ years of age [16, 18, 42, 43]. Additionally, prior studies have shown that bed-sharing is more likely to persist among youngsters who bed-share several times or more per week [6].

Tests of longitudinal associations found no evidence that reactive bed-sharing preceded or followed sleep disturbances and psychopathology. Thus, although bed-sharing in early childhood may be an indicator of sleep disturbances such as sleep terrors and difficulty staying asleep [15] and/or anxiety disorders such as separation anxiety, we found little evidence to suggest that bed-sharing is a cause or consequence of clinically significant problems.

### Strengths and limitations

This study had several strengths including a diverse, well-characterized sample, longitudinal design, and use of a structured diagnostic interview. There were also several limitations. First, all information about bed-sharing and psychopathology came from parent report and was derived from a set of items from the PAPA, an instrument which has good psychometric properties overall [44] and good test-retest reliability for bed-sharing frequency. However individual items were not compared to other metrics (bed-sharing questionnaire, direct observation or sleep diaries) for testing of concurrent validity. Second, our assessment of bed-sharing inquired about bed-sharing in response to child reluctance to sleep independently. Although some participating families may have engaged in bed-sharing for additional reasons, other reasons were not asked about in this study. Third, the study sample was ascertained in a single location in the Southeastern US that limits generalizability to the US population. Fourth, this longitudinal study could have been strengthened by assessing reactive and intentional bed-sharing, child sleep problems and psychopathology, parenting perceptions and practices (which might relate to subcultural differences), familial stress and parental psychopathology, and sociodemographics context particularly race and ethnicity in the same manner at multiple time points (e.g. infancy through early childhood) using a multi-method approach (i.e., parent report, direct measurement using actigraphs and/or video). These factors have been identified as central to understanding bed-sharing [24]. Then, guided by the transactional model of child sleep [21, 23], a bed-sharing model accounting for dynamic, bidirectional interactions between proximal child (e.g., child behavior at bedtime, sleep disturbances and anxiety) and parenting (e.g., maternal depression/anxiety, stress and parenting values/style) factors and distal, contextual factors (e.g., social/cultural norms in different raceialand ethnic groups and impact of socioeconomic status) could have been stipulated and tested. Current study findings suggest that future research could model the bidirectional and dynamic interrelations among child sleep disturbances and anxiety, the parenting practice of bed-sharing due to child reluctance to sleep alone, and variations in bed-sharing by race and ethnicity and socioeconomic factors during early childhood.

### Implications for clinical practice

In many respects, our findings suggest that bed-sharing in early childhood (2–6) is predominantly a normative, transient sleep practice influenced by sociodemographic context. Thus, when parents have questions or concerns about bed-sharing after infancy, pediatric providers can discuss with parents the evidence that bed-sharing is relatively common experience in this developmental period and one that may be influenced by family circumstances and cultural context. They can also note factors that may contribute to this increase in bed-sharing such as an increase in nighttime fears and heightened distress about being alone at night. Finally and perhaps most importantly, clinicians can reassure parents about the lack of association between preschool bed-sharing and clinically significant sleep or mental health problems as well as the likelihood that such bed-sharing will decrease, on its own, with time.

## Electronic supplementary material

**Supplementary material 1: Table S1**. Adjusted associations between Bed-Sharing Status and Sociodemographic Factors

## Data Availability

The datasets used and/or analyzed during the current study are available from the corresponding author on reasonable request.

## Abbreviations

AAP: American Academy of Pediatrics
PAPA: Preschool Age Psychiatric Assessment
