# Supplementary material for: Associations of preschool reactive bed-sharing with sociodemographic factors, sleep disturbance, and psychopathology
Source: Child Adolesc Psychiatry Ment Health. 2023 May 17;17:62. doi: 10.1186/s13034-023-00607-w (PMC10193615; doi:10.1186/s13034-023-00607-w)
Supplement: Supplementary file 1 — Supplementary material 1: Table S1. Adjusted associations between Bed-Sharing Status and Sociodemographic Factors [file 13034_2023_607_MOESM1_ESM.docx]

**Supplemental Table 1.**

| Table S1. Adjusted associations between Bed-Sharing Status and Sociodemographic Factors | | |
| --- | --- | --- |
|  | Nightly vs. none | Weekly vs. none |
|  | OR (95%CI), p | OR (95%CI), p |
|  |  |  |
| Age | **0.7 (0.6-0.9), <0.001** | **0.7 (0.6-0.9), 0.02** |
|  |  |  |
| % Male | 1.0 (0.6-1.8), 0.99 | 1.3 (0.7-2.6), 0.39 |
|  |  |  |
| % Hispanic | 1.5 (0.5-4.2), 0.47 | 1.0 (0.3-3.4), 0.94 |
| % Black | **3.4 (1.5-7.6), 0.003** | **4.6 (1.9-10.8), <0.001** |
| % White | -- | -- |
| % American Indian, Alaska Native or Asian | **4.7 (1.7-13.2), 0.002** | 3.1 (0.9-11.0), 0.07 |
|  |  |  |
| Family Income |  |  |
| Below Poverty | 0.9 (0.4-1.9), 0.69 | 0.6 (0.2-1.8), 0.36 |
|  |  |  |
| Highest Education |  |  |
| Less than HS | 1.8 (0.6-5.3), 0.31 | **0.2 (0.1-0.8), 0.03** |
| HS grad | 0.7 (0.2-1.9), 0.46 | 0.3 (0.1-1.2), 0.09 |
| Some college | 0.8 (0.4-1.8), 0.63 | 0.4 (0.2-1.0), 0.05 |
| College grad. | -- | -- |
